# Salutary effects of high-intensity interval training in persons with elevated cardiovascular risk

**DOI:** 10.12688/f1000research.8778.1

**Published:** 2016-09-07

**Authors:** Jerome L. Fleg

**Affiliations:** 1 Division of Cardiovascular Sciences, National Heart, Lung, and Blood Institute, Bethesda, MD, USA

**Keywords:** moderate-intensity continuous training, high-intensity interval training, cardiovascular

## Abstract

Although moderate-intensity continuous training (MICT) has been the traditional model for aerobic exercise training for over four decades, a growing body of literature has demonstrated equal if not greater improvement in aerobic capacity and similar beneficial effects on body composition, glucose metabolism, blood pressure, and quality of life from high-intensity interval training (HIIT). An advantage of HIIT over MICT is the shorter time required to perform the same amount of energy expenditure. The current brief review summarizes the effects of HIIT on peak aerobic capacity and cardiovascular risk factors in healthy adults and those with various cardiovascular diseases, including coronary artery disease, chronic heart failure, and post heart transplantation.

## Introduction

Aerobic exercise training has been extensively employed for several decades to improve functional capacity and cardiovascular (CV) risk factors in both healthy adults and those with cardiovascular disease (CVD). The traditional model for such training is typically a short (5–10 minute) warm up followed by 20–60 minutes of sustained moderate-intensity aerobic activity at 40–85% maximal aerobic capacity ^1^. Such programs employing moderate-intensity continuous training (MICT) typically increase peak oxygen consumption (VO _2_) by 10–30%, with greater improvements associated with lower baseline fitness, higher intensity exercise, and longer program duration ^1^. Additional benefits of MICT include modest blood pressure (BP) reduction, improved glucose and lipid metabolism, and mood elevation ^2^.

When performing aerobic exercise outside of a gym or cardiac rehabilitation (CR) program, individuals often vary the exercise intensity with short sprints as during a soccer or basketball game or in ascending hills on a bicycle. A program with such alternating short periods of high-intensity exercise at >85–90% peak VO _2_ interspersed among periods of lower intensity is known as high-intensity interval training (HIIT). Although HIIT has received relatively little attention as a therapeutic modality until the past decade, numerous recent reports have documented increases in peak VO _2_ and improvement in several CV risk factors similar to or greater than those observed with MICT. This brief review will summarize these salutary effects of HIIT in healthy adults, those with CV risk factors, and those with established CVD.

HIIT can be divided into two distinct subtypes. Sprint interval training (SIT) typically consists of 4–6 cycles of 20–40-second “all out” sprints followed by 4–5 minutes of recovery. Because of SIT’s extreme intensity, most SIT studies have been limited to healthy young individuals. In older populations and those with CVD, HIIT has been employed in the form of aerobic interval training (AIT), which is performed at lower intensities than SIT but for longer periods. A typical AIT protocol consists of 4–6 cycles, each comprising 4 minutes of exercise at 80–95% peak capacity followed by 3–4 minutes of recovery, performed on a treadmill or cycle ergometer ^3^. The current review focuses on studies that have employed the AIT form of HIIT, given its greater applicability to the general population and individuals with CV risk factors and CVD. An advantage of both SIT and AIT over traditional MICT is their shorter time requirement to achieve a similar energy expenditure.

## Animal studies

Aerobic exercise programs using HIIT in animal models have shown large improvements in peak VO _2_ similar or superior to those elicited by MICT ^4^. In addition, studies in mice and rats have demonstrated reductions in body mass, BP, and oxidative stress; augmented endothelial function; and improved metabolism in fat, liver, and skeletal muscle ^5, 6^. Of note, Wistar rats that underwent 5 consecutive days of HIIT on a treadmill up to 7 days before experimental ischemic reperfusion injury sustained 35–50% smaller myocardial infarctions (MIs) than did sedentary rats ^7^. A similar reduction in infarct size has been demonstrated after MICT protocols ^8^.

## Human studies

### Healthy adults

In healthy adults, a meta-analysis that included 723 healthy adults aged 18–45 years old from 28 controlled trials of 3–24 weeks’ duration showed a large absolute increase in peak VO _2 _of 4.9 mL/kg/minute following HIIT, which was greater than that seen with traditional MICT ^9^. Larger increases were observed among individuals with lower baseline fitness and in interventions longer than 10 weeks. Improved skeletal muscle mitochondrial function and pulmonary oxygen uptake kinetics have been demonstrated after only six HIIT sessions over 2 weeks in healthy untrained men ^10^. Although occasional studies have failed to show superior effects of HIIT over MICT on peak VO _2_, these studies generally employed a program duration shorter than 10 weeks ^3^. Because equivalent energy expenditure can be accomplished in less time with HIIT than with MICT, this may allow shorter exercise sessions with HIIT, thereby improving adherence.

### Obesity

Given the frequent association of obesity with low fitness and with CV risk factors such as hypertension, glucose intolerance, and dyslipidemia, a strong rationale exists for aerobic exercise training in such individuals. In three studies 3–6 months in duration, similar improvements in body mass index and waist circumference were observed in overweight/obese participants following both MICT and HIIT ^11– 13^. The equivalent anthropometric changes might be attributed to the similar total energy expenditure with the two programs in those studies.

### Hypertension

Traditional MICT exerts significant reduction of BP in hypertensive adults, especially those with suboptimal baseline BP control ^14^. In such individuals who were not receiving antihypertensive drugs, HIIT for ≥12 weeks appears to provide a similar antihypertensive effect ^3^. For example, 16 weeks of MICT or HIIT led to similar reductions of systolic (10 mmHg) and diastolic (6 mmHg) BP in middle-aged adults with elevated BP at baseline ^13^. A 12-week HIIT program in overweight/obese adolescents with borderline hypertension also elicited significant reductions in both systolic BP (9 mmHg) and diastolic BP (6 mmHg) ^15^.

### Lipids

Among individuals with dyslipidemia, traditional MICT typically elicits modest increases in high-density lipoprotein cholesterol (HDL-C) but exerts no consistent effect on low-density lipoprotein cholesterol or triglycerides ^16^. Similar effects on blood lipids are observed with HIIT ^17^. Modest increases of HDL-C with HIIT required 8 or more weeks of training and occurred in only 3 of 10 such studies ^17^. Given these inconsistent effects on HDL-C without benefit on other lipid fractions, neither HIIT nor MICT can generally be recommended as primary therapy for the treatment of dyslipidemia.

### Glucose metabolism

Substantial evidence suggests that HIIT improves glucose metabolism to a similar or greater extent than does MICT. Augmented insulin sensitivity occurs as early as 2 weeks after the initiation of HIIT ^18^ and has been shown in both healthy adults ^19^ and those with metabolic syndrome ^13^. Improved oral glucose tolerance test results have been consistently reported after HIIT ^3, 18, 20^. Fasting blood glucose did not change in studies of less than 12 weeks’ duration but declined in four of seven longer studies ^3^. Small studies have found improved glycemic control in patients with type 2 diabetes ^21– 23^.

### Adults with heart disease

Incontrovertible evidence for the benefits of MICT in patients with CVD has accumulated over the past four decades through hospital-based CR programs involving patients after acute MI, coronary revascularization, valvular surgery, and those with chronic heart failure (CHF). Similar, though less extensively studied, salutary effects of HIIT have been demonstrated in patients with CVD and are reviewed below.

***Coronary artery disease.*** Multiple studies have documented the benefit of HIIT in patients with coronary artery disease (CAD), the most prevalent form of heart disease in developed countries. A meta-analysis of 229 patients with CAD demonstrated a 1.5 mL/kg/minute larger mean increase in peak VO _2_ with HIIT than with MICT ^24^. A larger meta-analysis that included 472 CAD patients showed a similarly augmented gain in peak VO _2_ (1.8 mL/kg/minute mean difference) compared with MICT ^25^. In this latter analysis, no differences in blood glucose or lipids were observed between the groups. However, resting heart rate (-1.8 beats/minute) and body weight (-0.5 kg) declined slightly after MICT but not HIIT. A recently published randomized trial also showed a significantly larger increase in peak VO _2_ after 8 weeks of HIIT compared to MICT (4.5 versus 2.5 mL/kg/minute), respectively ^26^.

***Chronic heart failure.*** Although CR was not approved until 2014 by the Center for Medicare and Medicaid Services for patients with CHF, considerable evidence has accrued on the benefits of HIIT in this population. One of the first studies to examine the effect of HIIT in CHF patients showed a remarkable 46% increase in peak VO _2_ in nine older patients (mean 75 years) with post-MI CHF versus an increase of only 14% with MICT ^27^. Favorable left ventricular remodeling and increased left ventricular ejection fraction occurred only after HIIT. Three recent meta-analyses have confirmed a larger increase in peak VO _2_ with HIIT than MICT in CHF patients ^28– 30^. Haykowsky *et al.* ^28^ showed a mean 2.1 mL/kg/minute larger increase in peak VO _2_ with HIIT in seven studies encompassing 168 patients. In a meta-analysis by Smart *et al.* ^29^ involving 212 patients who performed HIIT and 66 who performed MICT, the mean improvement in peak VO _2_ was 1.0 mL/kg/minute larger after HIIT. A very large meta-analysis that included 74 exercise-based CR trials involving 5877 participants showed a 23% mean increase in peak VO _2_ among patients receiving HIT versus a 13% increase in those undergoing MICT ^30^. Of note, withdrawal from the CR program was lower with HIIT. It should be noted that all of the studies and meta-analyses cited above excluded patients with CHF and preserved ejection fraction (HFpEF), who comprise 30–40% of the CHF population. Studies of HIIT are clearly needed in this latter group.

***Heart transplant recipients.*** Although data remain limited regarding the effects of HIIT in heart transplant recipients, available evidence suggests a similar benefit as seen with MICT. Compared to a usual care control group, 24 stable heart transplant recipients 1–8 years post transplantation demonstrated a 3.6 mL/kg/minute higher peak VO _2_ after 1 year of HIIT ^31^. In a study of 16 patients ≥12 months post transplantation, 8 weeks of HIIT induced a larger increase in peak VO _2_ than did MICT (4.9 versus 2.6 mL/kg/minute) ^32^.

### Quality of life

Although quality of life (QOL) is often considered a “softer” outcome variable than changes in physiological indices, it is probably a more relevant variable to patients. In this context, both MICT and HIIT have elicited improvement in QOL in a variety of clinical settings. For example, improvement in QOL after HIIT has been reported in patients with CAD ^26^ and CHF ^33^, cardiac transplant recipients ^32^, and generally healthy older adults ^34^. Such improvement in QOL may reinforce the patient’s motivation to continue exercise participation.

### Safety of HIIT

An important concern in prescribing any exercise program, especially in older adults and patients with CVD or other comorbidities, is safety. To date, the evidence suggests that HIIT is well tolerated to a similar extent as MICT. In the large meta-analysis of CHF patients previously mentioned, no deaths were attributed to exercise training regardless of intensity ^30^. A similar finding was reported in an analysis of 4846 CAD patients from three Norwegian CR centers ^35^. In this study, major complications were one per 129,456 hours of MICT and one per 23,182 hours of HIIT. Nevertheless, HIIT may not be appropriate for frail older adults, those with significant orthopedic, neurological, or balance difficulties, those with marked obesity, or those with severe deconditioning. In such individuals, an initial low-to-moderate-intensity exercise program appears to be preferable.

### Mechanistic insights

Given the substantial benefits of HIIT in a wide variety of settings, insights into the biochemical and molecular adaptations accompanying HIIT are worthy of inquiry. In patients with obesity, metabolic syndrome, or CHF, HIIT induced increases in peroxisome proliferator-activated receptor γ co-activator (PGC)-1α, a master regulator of oxidative phenotype ^12, 13, 27^ and muscle mitochondrial capacity ^10, 18^. In addition, sarcoplasmic reticulum calcium uptake was augmented by HIIT but not MICT, likely owing to an increased ability to perform high-intensity muscle contractions ^12, 18, 25^. Another salutary effect of HIIT is to enhance flow-mediated dilation of the brachial artery, a measure of vascular endothelial function mediated in part by increased nitric oxide availability ^12, 13, 27^. Finally, increased cardiac stroke volume has been reported after HIIT in obese women ^36^, sedentary middle-aged adults ^37^, and patients with CHF ^27^.

## Conclusions

Although less thoroughly investigated than traditional aerobic training programs using MICT, HIIT appears to share similar salutary effects, with generally larger increases in peak VO _2_, without a significant difference in serious adverse events in appropriately selected individuals. Future studies should explore the long-term effects of HIIT on health outcomes as well as its effects when combined with dietary, drug, and cardiac device interventions in patients with CV disease.

## Disclaimer

The views expressed are those of the author and do not necessarily represent those of the National Heart, Lung, and Blood Institute or the U.S. Department of Health and Human Services.
